# The Relationship Between Public Service Motivation and Affective Commitment in the Public Sector Change: A Moderated Mediation Model

**DOI:** 10.3389/fpsyg.2021.631948

**Published:** 2021-06-18

**Authors:** Sirui Sun

**Affiliations:** School of International and Public Affairs, Shanghai Jiao Tong University, Shanghai, China

**Keywords:** public sector change, affective commitment to change, public service motivation, superficial harmony, voice behavior

## Abstract

How can public organizations promote change recipients’ affective commitment to public sector change? Based on socially desirable responding theory, this study explores the theoretical mechanism and boundary effect of the relationship between public service motivation and affective commitment to change. By conducting a survey of 465 front-line public employees in an eastern Chinese city undergoing public sector change, this study found that voice behavior partially mediates the relationship between public service motivation and affective commitment to change. Superficial harmony also negatively moderates the relationship between public service motivation and affective commitment to change through the mediation of voice behavior. This study mainly contributes to our understanding of the theoretical mechanism and the conditional effect of change recipients’ affective commitment during public sector change.

## Introduction

In recent decades, to keep pace with the rapid economic changes of globalization, public organizations have faced great pressure relating to organizational change (Homberg et al., 2019; Ahmad et al., 2020a). Many management studies notice that the decisive way to succeed with organizational change is with the proactive support of change recipients (Oreg et al., 2018; Ahmad et al., 2020a). However, compared to learning why public sector change affects those recipients differently, as private sector management researchers do in that context (Oreg, 2006), public administration scholars are more interested in issues from a macro perspective, such as reform design, structural adjustment, and reform-effect evaluation (Kiefer et al., 2014; Ahmad et al., 2020b). But frontline public employees implement the actual public sector changes; hence, the extent to which public employees proactively engage in organizational change is also essential for its success (Kelman, 2005; Hassan et al., 2020). Affective commitment to change is a proactive support response, defined as “a desire to support change with an inherent belief in change” (Meyer et al., 2002, p. 19). It largely reflects the extent to which individuals proactively engage in the change (Oreg et al., 2018). In this study, we focus on the theoretical mechanism of affective commitment to change and add the theoretical values of change proactive support behavior in the context of public administration studies.

The body of literature on affective commitment to change among public employees shows that it has grown at a modest rate (Wright et al., 2013; Homberg et al., 2019), and the antecedents and consequences of affective commitment to change within the public sector desperately deserve more attention (Wright et al., 2013; Ahmad and Cheng, 2018; Oreg et al., 2018). We take a step in this direction by getting the whole picture of why and when the individuals could generate affective commitment to change to public sector change. To develop our model, we draw on public service motivation theory as one of the most important motivation mechanisms that explain the prosocial behavior of public sector employees (Perry and Wise, 1990) that has attracted scholars’ attention during the past 30 years (Perry and Vandenabeele, 2015). Previous studies identify public service motivation as an important personal dispositional factor that relates positively to such a positive change-related response as affective commitment to change (Wright et al., 2013; Liu and Zhang, 2019; Hassan et al., 2020). Although past research pays much attention to public service motivation and affective commitment to change, we know very little about the theoretical mechanisms that may underlie this relationship (Wright et al., 2013; Hassan et al., 2020). Moreover, we lack empirical evidence for understanding this question in an eastern, Asian country largely affected by Confucius’s culture (Leung et al., 2011), an implicit culture that opposes western countries’ initiative and sense of spiritual adventure(Ahmad and Cheng, 2018).

To address this gap in the literature, this study attempts to explore a mediated-moderation model of the relationship between public service motivation and affective commitment to change in an eastern Chinese city undergoing public sector change. Based on social desirability theory, we believe that self-deceptive enhancement and impression management are two distinct ways that individuals relate to organizational change (Lalwani et al., 2009; Zhang and Wei, 2017). Self-deceptive enhancement is “the tendency to describe oneself in an inflated yet honestly held manner and to see oneself in a positive, overconfident light” (Lalwani et al., 2009, p. 2). Voice behavior is an informal, arbitrary communication (Liang et al., 2012). Weiss and Morrison (2019) propose that individuals exert their voice behavior by showing their ability or expressing their willingness to help others (Weiss and Morrison, 2019). We argue that people who can express their views in a changing environment also have strong self-deceptive enhancement motives. Also, people who are willing to voice, means they believe in their ability and willingness to point out the problems with change, hence, strengthening their honesty to the organizational change. However, in China’s specific cultural atmosphere, especially with public sector change as a typical conflict scenario, another impression-management motive, reflected as maintaining superficial harmony (Zhang and Wei, 2017), makes them dissimulate or be silent about change, rather than proactively show support. High-level superficial-harmony individuals fear that showing support will lead to interpersonal conflict and undermine the positive impression others have of them, so they avoid conflicts during public sector change. Hence, we conduct that the superficial harmony could be the conditional effect.

Therefore, to close the knowledge gap, this study develops a more complete model to explore the relationship between public service motivation and affective commitment to change. The theory of two types of socially desirable responding explains voice behavior as the mediator and superficial harmony as a boundary effect in the relationship with public service motivation. In short, this study provides a rich theoretical explanation for the change-proactive support of public employees in public sector change, especially for the country affected by Confucius’s culture.

## Theory and Hypotheses

### Affective Commitment to Change and Socially Desirable Responding Theory

Although much research focuses on the resistance of change recipients in the long term, little research exists on the positive reactions of change recipients, especially affective reactions (Oreg et al., 2011). Affective commitment to change often reflects a desire to provide support for change, with an intrinsic sense of belief in change (Herscovitch and Meyer, 2002, p. 475). Individuals with a high-level affective commitment to change believe that what they are doing is valuable and necessary to help the organization make the change successful (Meyer et al., 2007). Previous studies find that employees’ affective responses to change play a crucial role in their recognition and support for the change (Parish et al., 2008). So, in recent studies, scholars argue that affective commitment to change is individual proactive support that should be strongly encouraged during organizational change (Oreg et al., 2018). Hence, as an emotional embodiment, affective commitment to change is most worthy of scholars’ attention (Meyer et al., 2007; Oreg et al., 2018). In this study, we define affective commitment to change as one’s favorable intention regarding current social norms and standards of organizational change (Zerbe and Paulhus, 1987). Moreover, proactively involving intention toward change is socially desirable for public-sector managers, especially in an environment where public sector change lacks support.

Researchers argue that the establishment of affective commitment to change closely relates to many internal and external factors, such as individuals’ expected belief in change, management of the change process, and employment quality in the period of change (Jimmieson et al., 2008; Kim et al., 2011; Rafferty and Minbashian, 2019). We lack the empirical study to explain the theoretical mechanism of deriving affective commitment to change from a socially desirable responding theory that entails “the tendency of individuals to present themselves favorably concerning the norms and standards of the society”(Zerbe and Paulhus, 1987, p. 1). Previous studies maintain that individuals tend to present themselves in socially desirable ways while fearing negative evaluations or social rejection by others (Zhang and Wei, 2017). Distinguishing two types of socially desirable responses (self-deceptive enhancement and impression management) could add to our understanding of individualism-collectivism (Lalwani et al., 2009). We regard the affective commitment to change as a typical favorable response, and this study’s main focus is the antecedents reflected as self-deceptive enhancement and impression-management characteristics. We examine the balance of individualism-collectivism cultural values in responding to the socially desirable change supportive intention. The following section clarifies the theoretical considerations of this study.

### Public Service Motivation and Affective Commitment to Change

Organizational change easily leads employees to feel suspicious and stress reduces their affective commitment to change (Elias, 2009). However, previous studies have found that employees with high levels of public service motivation foster them regarding change as the process of improving government services and benefiting the public, thus promoting high levels of affective commitment to change (Naff and Crum, 1999; Wright et al., 2013). Public service motivation is “the motivational force that induces individuals to perform meaningful public, community, and social service”(Brewer and Selden, 1998, p. 417). People with high-level public service motivation have a greater sense of mission and responsibility at work (Pattakos, 2004). Affected by public service motivation, public employees have a sense of commitment to public service, characteristics of benevolence, the expectation of serving others, and a strong determination to serve the community (Houston, 2006).

Public sector studies have largely examined the positive relationship between public service motivation and affective commitment to change (Wright et al., 2013; Van der Voet et al., 2016; Hassan et al., 2020). This study holds that public service motivation is oriented toward serving society and others and influenced by this motivation, individuals are more likely to express prosocial behavior and willingly support organizational change (Ahmad et al., 2020c). On the one hand, such individuals want the organizational change to deliver meaningful public service better. On the other hand, they agree with and are loyal to the organization’s decision-making, so they see organizational change as the instrument of advancing the public sector. Thus, their affective commitment to change is higher.

### The Mediating Effect of Voice Behavior

Public service motivation significantly and positively correlates with individual job satisfaction, organizational commitment, and organizational citizenship behavior (Perry and Vandenabeele, 2015). Individuals with higher public service motivation show more positive behaviors, attitudes, and job performance because they cherish the rare opportunity to work in the public sector and serve the public (Perry and Wise, 1990; Liu et al., 2015). As a result, organizational problems during the change process can easily arouse the attention of more highly motivated public employees, igniting them to resolve the problems or speak out about potential issues (Crant et al., 2011). In the context of organizational change, employees are willing to voice, indicating their awareness of the knowledge and ability that help them to perform their duty (Weiss and Morrison, 2019). Even though they might take interpersonal risks and performance appraisal losses, this closely relates to the public service dimension of self-sacrifice (Perry and Hondeghem, 2008; Wright et al., 2013). Voice is essentially a proactive employee behavior that helps bring about changes in organizations (Parker and Collins, 2010). As a proactive behavior, the most important driving force of voice behavior is motivation (Parker et al., 2010). A unique prosocial motivational base for public employees, public service motivation embodies employees’ spirit of the “public servant,” well-suited to motivating them. Specifically, public service motivation closely relates to individuals’ positive behavior performance, job performance, and extra-role behavior (Liu et al., 2015). Hence, we believe that individuals with high-level public service motivation are more willing to express themselves vocally during an organizational change.

Voice behavior is a proactive employee behavior (Liang et al., 2012) that could make a situation better or challenge an existing situation (LePine and Van Dyne, 1998), particularly voice behavior that emphasizes flexibility, innovation, and continuous improvement (Howard, 1995). Voice is one of the most important manifestations of an employee’s active involvement in the work because it challenges authority by providing useful suggestions or questions (Morrison, 2014). Therefore, we believe that in the context of organizational change, people who are willing to vocalize have stronger motives of self-deceptive enhancement. Voice behavior allows them to better demonstrate the change-support intention that organizations expect, their ability to exercise control in the uncertainty of the changing environment, and their desire to improve the organization.

Voice behavior requires resources, and only when employees have access to resources do they have an environment in which to express their voice (Kim et al., 2019). In other words, people able to voice have more resources in the organization than ordinary employees, and the socially desirable behavior, such as affective commitment to change, is more likely to generate this kind of important status for public employees. Voice behavior is often defined as a bottom-up process aimed at improving standard procedures (Walumbwa and Schaubroeck, 2009). As an important extra-role behavior, voice behavior reflects inconsistency with specific organizational behavior and ethics. It also indicates employees’ concerns about potential problems in the organization or their willingness to improve (Walumbwa and Schaubroeck, 2009). As a result, voice behavior also can appear to indicate people more committed to organizational change. People with a high-level affective commitment to change show a greater likelihood of contributing to the organization’s development goals, willing to be highly coordinated and supportive of the change (Parish et al., 2008). Voice behavior suggests that employees willing to offer advice are more attuned to achieving organizational goals than fearing personal risks (Crant et al., 2011). Voice behavior is a positive behavior in which employees proactively participate during work-related decision-making, reflecting their support for the change. Therefore, we believe that voice behavior positively affects employees’ affective commitment to change, and that voice behavior could link public service motivation and affective commitment to change; thus, we propose Hypothesis 1.

**Hypothesis 1: Voice behavior mediates the relationship between public service motivation and affective commitment to change.**

### The Moderating Effect of Superficial Harmony

Interpersonal harmony theory originated from Chinese culture and argues that approaching disagreements with interpersonal harmony is a guiding principle due to Confucian influence (Leung et al., 2011; Zhang and Wei, 2017). Harmony theory is a fundamental explanation of how the Chinese cope with conflicts (Chang, 2001), and it has two constructs: genuine harmony and superficial harmony (Leung et al., 2011). Superficial harmony is “associated with an instrumental motive, as it focuses on the negative consequences and backlashes of a relationship” (Leung et al., 2011, p. 2). When a sense of commitment is low, and tolerance is high, superficial harmony forms (Das and Kumar, 2009). The expression of harmony with defensive motives (i.e., to avoid division) is superficial harmony, which holds that maintaining a smooth interpersonal relationship protects self-interest. There is no need to take various inconsistent actions that hinder and undermine one’s interests (Lim, 2009; Leung et al., 2011). The present study asserts that superficial harmony reflects people’s impression-management motive toward socially desirable responding to public sector change, and superficial harmony influencing all individual’s intentions would become more conservative and cautious, especially during organizational change.

The value of superficial harmony reflects a defensive relationship motivation (Zhang and Wei, 2017). The harmonious value of social expectations allows individuals to protect themselves from unnecessary social punishments (Leung, 1997). Due to the influence of superficial harmony values, the positive influence of public service motivation on voice behavior will weaken as employees worry that taking proactive action will cause conflicts with colleagues (Detert and Trevino, 2010). Oriented by superficial-harmony values, people tend to choose mutually agreeable behaviors that maintain harmony. Voice is a dangerous activity; risk includes that the leader does not accept advice (Detert and Edmondson, 2011), and, considering the need for impression management, they would not engage in such risky behavior as voicing.

To establish a good image as socially expected, superficial-harmony value makes it possible to weaken the relationship between public service motivation and voice behavior. In an uncertain environment, people must have interpersonal harmony to maintain certainty; social ties will help them better adjust and cooperate in the face of organizational change, and superficial-harmony values are a strategy to avoid interpersonal conflict and maintain social relationships (Zhang and Wei, 2017). However, allowing employees to vocalize on organizational change will increase interpersonal conflicts. Even though the people with high-level public service motivation want to vocalize under the influence of superficial-harmony considerations, they would not do so. Specifically, voicing might cause additional colleague conflict and is not worth disrupting healthy social relationships. Employees with high-level public service motivation could deliver public service smoothly during the change with colleagues’ help, so they will not proactively vocalize and risk undermining harmonious work in times of change.

In addition, superficial harmony is a value that ostensibly emphasizes the harmonious state of groups but is genuinely concerned with personal interests (Leung et al., 2011). We maintain that the occurrence of such dissonance will negatively affect an individual’s affective commitment in the context of change. Public service motivation promoting voice behavior is a process in which public employees speak out about organizational problems and reflect a genuine affective commitment to the change, showing their self-sacrifice and strong organization-oriented motives. Individuals with a strong sense of superficial harmony have more concerns about the overall stability and preservation of their interests than with the success of the organizational change. So, voice or not depends on whether the organizational change will harm them, if it does, they would not voice and show their support to the change. Therefore, their affective commitment to change is lower. In conclusion, we believe that people with a strong sense of superficial harmony are less likely to proactively participate in activities that require them to challenge authority in the context of change, and this shows their lesser loyalty to the change. On the contrary, people with a weaker sense of superficial harmony are more likely to vocalize, based on the pursuit of efficient public services, and this shows a higher level of affective commitment to change. Consequently, we propose Hypothesis 2:

**Hypothesis 2: Superficial harmony will negatively moderate the relationship between public service motivation and affective commitment to change through a voice-behavior mediator.**

## Materials and Methods

### The Context of the Study

The survey was conducted in a city in Eastern China. The respondents were grassroots government workers. At the end of October 2017, this unit was carrying out institutional restriction reform while we were conducting the research. The employees participating in this research worked for the district’s bureau-level public institutions before the reform. After the reform, their employment relationship was translated to a lower level-grassroots government; as a result, lower-level institutional management limits their pay and promotions in the long run. In this context of organizational change, managers urgently need to understand how to motivate the employees and have made improvements in human resources management practice. The researchers mainly provided such human resource management consulting services for this public sector unit during the change, and then collected all data.

A total of 510 employees who had experienced the above-mentioned reforms participated in our survey. The questionnaire was translated into Chinese through standard translation and back-translation procedures. After screening unqualified questionnaires, 465 valid questionnaires were retained, with a response rate of 91.18%. In total there were 381 male respondents, accounting for 81.94% of responses. All participants were aged between 23 and 60, with an average age of 40.94 years. In total, 364 were undergraduates, accounting for 78.28% and 65.81% were civil servants.

### Measures

All questionnaires in this study were measured using a Likert six-point scale with scores ranging from 1 to 6, representing personal opinions ranging from “strongly opposed” to “strongly agreed.”

#### Public Service Motivation

This study used the 5-item scale used by Pandey et al. (2008). A sample item was “meaningful public services are essential to me.” The Cronbach’s Alpha was 0.909.

#### Voice Behavior

The 10-item voice behavior scale used was developed by Liang et al. (2012). A sample item was “I will take the initiative to speak reasonable suggestions to help achieve the organization goal.” The Cronbach’s Alpha was 0.81.

#### Affective Commitment Change

The 4-item used were taken from Herscovitch and Meyer’s (2002) scale of affective commitment to change. The sample item was, “I believe this change is valuable.” The Cronbach’s Alpha was 0.81.

#### Superficial Harmony

This study used the 8-item scale developed by Leung et al. (2011) to measure superficial harmony. The sample item was, “When others are more powerful than yourself, you must be patient with them.” The Cronbach’s Alpha was 0.90.

#### Control Variables

In this study, we controlled demographic variables, such as gender, age, education background, and tenure. Previous studies verify that these variables can influence affective commitment to change (Walker et al., 2007).

## Results

### Measurement Models

This is a cross-sectional, self-reported survey, and all the variables are defined in the individual group, so we examined common method bias first. Using the Harman single factor test, the results showed that the unrotated explained variance of the first factor was 31.2%, lower than 40%, indicating the common method bias in this study is not serious (Podsakoff et al., 2000). Then, to ensure the discriminative validity of affective commitment to change, superficial harmony, public service motivation, and voice behavior, we conducted confirmatory factor analysis (CFA) to examine different models. As Table 1 shows, the four-factor model this study proposed is good (χ^2^ = 1401.2, *df* = 293, χ^2^/*df* = 4.78, RMSEA = 0.09, CFI = 0.86, TLI = 0.95, SRMR = 0.07), whereas all the three-factors, two-factors, and one-factor models did not fit well, demonstrating that the four constructs have good distinctiveness.

**TABLE 1:** The confirmatory factor analysis results.

	**χ^2^**	**df**	**RMSEA**	**CFI**	**TLI**	**SRMR**	**Model**	**△χ^2^/**△df****	**△CFI**
1. Null model	8445.07	325							
2. One factor model	4631.71	299	0.18	0.47	0.42	0.17			
3. Model 1	1401.2	293	0.09	0.86	0.95	0.07			
4. Model 2: PSM + SH	2937.67	296	0.14	0.68	0.64	0.15	2 vs. 1	512.16	0.18
5. Model 3: PSM + VB	2261.75	296	0.12	0.76	0.73	0.09	3 vs. 1	286.85	0.10
6. Model 4: PSM + ACC	2178.37	296	0.12	0.77	0.75	0.09	4 vs. 1	259.06	0.09
7. Model 5:VB + ACC	2252.02	296	0.12	0.76	0.74	0.09	5 vs. 1	283.61	0.10

*Model 1, including all four variables. PSM, public service motivation; SH, superficial harmony; VB, voice behavior; ACC, affective commitment to change.*

Table 2 shows all the standardized factor loadings of CFA and all the standardized factor loadings that exceeded the threshold value of 0.4. The CR values for four factors were greater than 0.8, which indicated acceptable construct reliability. The AVE values for the four factors were greater than 0.5, which indicated the adequate convergent validity of the constructs.

**TABLE 2:** Standardized factor loading of confirmatory factor analysis for the CFA sample.

**Constructs/Items**	**Mean**	**SD**	**λ**	**AVE**	**CR**	**Constructs/Items**	**Mean**	**SD**	**λ**	**AVE**	**CR**
Public service motivation				0.74	0.93	Voice behavior				0.55	0.92
PSM1	4.51	1.23	0.83			VB1	3.99	1.06	0.74		
PSM2	4.58	1.15	0.86			VB2	4	1.05	0.76		
PSM3	4.46	1.18	0.89			VB3	4.13	1.02	0.85		
PSM4	4.28	1.25	0.86			VB4	4.3	1.07	0.87		
PSM5	4.33	1.17	0.85			VB5	4.21	1.02	0.88		
Superficial harmony				0.59	0.92	VB6	4.3	0.98	0.9		
SH1	3.19	1.42	0.68			VB7	4.36	0.95	0.87		
SH2	3.05	1.98	0.79			VB8	4.37	2.05	0.43		
SH3	3.56	1.3	0.78			VB9	4.4	2.09	0.42		
SH4	3.45	1.37	0.78			VB10	4.38	2.47	0.4		
SH5	3.35	1.37	0.84			Affective commitment to change				0.64	0.87
SH6	3.45	1.34	0.78			ACC1	4.23	2.23	0.52		
SH7	2.53	1.44	0.75			ACC2	3.98	1.21	0.89		
SH8	3.34	1.34	0.76			ACC3	4.1	1.49	0.83		
						ACC4	3.93	1.29	0.89		

**N* = 465. PSM, public service motivation; SH, superficial harmony; VB, voice behavior; ACC, affective commitment to change. **λ** = standardized factor loading, CR, construct reliability; AVE, average variance extracted. All factor loadings were statically significant at *p* < 0.05.*

### Descriptive Statistics

Before using the structural equation model to test the hypotheses, we first performed a correlation analysis among research variables. According to the results in Table 3, the correlation coefficients between public service motivation, voice behavior, and affective commitment to change reached a significant value.

**TABLE 3:** Means, standard deviations, and correlations.

	**Mean**	**SD**	**1**	**2**	**3**	**4**	**5**	**6**	**7**	**8**
1. Age	40.94	8.38								
2. Sex	1.18	0.38	−0.15**							
3. Edu	1.81	0.43	−0.36**	0.05						
4. Tenure	10.74	7.21	0.65**	−0.16**	−0.24**					
5. PSM	4.43	1.03	0.02	0.10*	0.02	–0.09	–			
6. VB	4.25	0.91	0.06	0.09	0.02	–0.08	0.56**	–		
7. ACC	3.88	1.22	–0.04	0.04	–0.01	−0.11*	0.41**	0.36**	–	
8. SH	3.23	1.06	–0.06	0.02	–0.08	–0.09	−0.17**	–0.02	0.07	–

*N* = 465. *p < 0.05. **p < 0.01(Two-tailed test). Variables 5–8 are measured on a Six-point Likert-type scale. Gender was coded as male = 1, female = 2. Educational background was coded as junior college = 1, bachelor = 2, master = 3, doctor = 4. PSM, public service motivation; SH, superficial harmony; VB, voice behavior; ACC, affective commitment to change.

### Hypothesis Testing

We used the structural equation model to test our hypothesis and to get the model fit result. We constructed two models to compare with the fitting index and examined the mediation effect. The theoretical model (voice behavior is the meditator) shows a good model fit (χ^2^ = 11.97, *df* = 6, χ^2^/*df* = 2, RMSEA = 0.05, CFI = 0.97, TLI = 0.93, SRMR = 0.02). The competitive model (no meditator in the model) does not fit well (χ^2^ = 41.99, *df* = 7, χ^2^/*df* = 6, RMSEA = 0.12, CFI = 0.85, TLI = 0.67, SRMR = 0.04). Hence, the result of model fitting shows that voice behavior is the mediator of public service motivation and affective commitment to change. The data supported Hypothesis 1.

**FIGURE 1:**
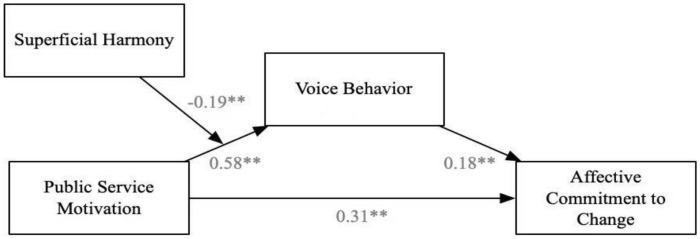
Results of theorical model. **p < 0.01.

Figure 1 shows the result of the path coefficient of the theoretical model. After controlling the control variables, such as gender (β = −0.03, *n.s.*), age (β = −0.08, *n.s.*), education background (β = −0.07, *n.s.*), and tenure (β = −0.04, *n.s.*), the relationship between public service motivation and voice behavior is significant (β = 0.58, *p* < 0.01); the confidence interval (CI) was [0.49, 0.65]. The relationship of voice behavior to affective commitment to change is significant (β = 0.18, *p* < 0.01); CI was [0.07, 0.29]. The direct relationship from public service motivation to affective commitment to change is also significant (β = 0.31, *p*<0.01); CI was [0.2, 0.42], indicating that the voice behavior is a partial mediation, and Hypothesis 1 was supported. Subsequently, we examined the moderating effect. The results in Figure 1 show that the interaction between superficial harmony and public service motivation had a significant effect on the affective commitment to change through voice behavior (β = −0.19, *p* < 0.05); CI was [−0.33, −0.04], Hypothesis 2 was supported.

**FIGURE 2:**
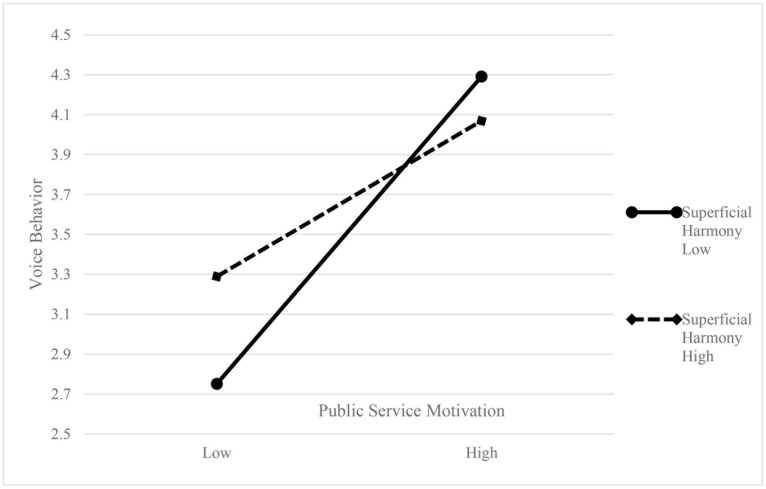
The moderating effect of superficial harmony on the relationship between public service motivation and voice behavior.

To test the mediated moderation effect further, we followed Edwards and Lambert’s (2007) method, using Mplus 7.4 software to perform path analysis, and ran the theoretical model in low (mean −1 SD), and high (mean +1 SD) moderating context. Bootstrapping 10,000 times samples was used to compute bias-corrected confidence intervals; Table 4 shows the results. In the high superficial-harmony group, the indirect effect was significant (β = 0.36, *p* < 0.01), CI was [0.19, 0.49]. In the low superficial-harmony group, the indirect effect was also significant (β = 0.43, *p* < 0.01), CI was [0.30, 0.53]. The difference between the two groups was significant (*p* < 0.1). Therefore, Hypothesis 2 was further supported. Figure 2 shows the moderating effect.

**TABLE 4:** Indirect effects test of different conditions.

**Effects**	**Level of superficial harmony**	**B**	**SD**	**95% Confidence interval**	
				**Low**	**High**
Indirect effects	High	0.36	0.09	0.19	0.49
	Low	0.43	0.07	0.30	0.53
	Group Differences	−0.06	0.04	−0.14	−0.02

*Bootstrap = 10,000; *N* = 465.*

## Discussion

### Research Conclusions

Based on socially desirable responding theory, this study examined the theoretical mechanism and boundary effect of affective commitment to change in public sector change. The data supported the two hypotheses. Specifically, this research finds that: (1) Voice behavior partially moderates the relationship between public service motivation and affective commitment to change; (2) Superficial harmony negatively mediates the relationship between public service motivation and affective commitment to change through the mediation of voice behavior. These two findings help us address the numerous calls to discover theoretical mechanisms of proactive change response under the same change context, such as the public sector (Oreg et al., 2018), promoting researchers involving individual behavior studies in the public administration field (Kelman, 2005; Wright et al., 2013; Ahmad et al., 2020d).

### Theoretical Contributions

This research has made the following theoretical contributions. First, this study links the proactive change support behavior research into the public administration field. This study builds a moderated-mediation model between public service motivation and changing support intention in the Chinese public sector, responding to the call to understand this effect in various public organizations in different countries (Ahmad et al., 2020a; Hassan et al., 2020). Our study underscores the critical roles of public service motivation regarding encouraging change-related intention in an Eastern Asian context. Notably, the present study also reveals why and when public service motivation can encourage affective commitment to change.

Second, the study advances the literature by introducing two types of socially desirable responding embedded model of affective commitment to change. Previous studies focus on theoretical mechanisms of affective commitment to change derived from the theory of planned behavior (Ahmad et al., 2020b, d). This study proposes the behavioral mechanism and value perspective to emphasize why socially desirable change commitment intention is generated. This helps us to advance the understanding of the whole picture of theoretical mechanisms of affective commitment to change.

Finally, the study specifies the research context of the public sector influenced by Confucianism, a relatively novel perspective, and an important theory integration (Lalwani et al., 2009; Zhang and Wei, 2017). By discussing superficial harmony as a value concept derived from the development of traditional Chinese culture (Leung et al., 2011), we can see how it affects the Chinese public employee’s work attitude and behavior. This will add a meaningful attempt to embed cultural influence in management studies, especially for long-term focus on the system, policy, and structure-related public administration studies.

### Managerial Implications

The implications for management are as follows. First, the findings of this study suggest that to encourage individuals to treat change proactively, the managers should provide a friendly environment for employees to voice and even communicate freely to their supervisors during times of organizational change. Second, the initial step in addressing such problems as “not taking charge” and “inaction” in the Chinese public sector is to avoid the bad influence “ripple” from an individual’s superficial harmony. Officials should form the reconstruction of human resource management practice, communication mechanisms, or even the legal system, to block the spread of this unhealthy atmosphere. Finally, our results show that the public sector should recruit and cultivate more high-level public service motivation practitioners, to make the public service sector better deliver meaningful public service to society.

### Limitations and Future Research

First, all the survey variables in this study were summarized at the same level. However, the influence from the team levels on employees’ affective commitment to change is also important; perhaps a future study could examine the organizational-level factors much more deeply. Second, the research still has common method variance since all the data were self-reported, raising the hope a longitudinal study conducted in the future will address the cross-sectional design problems. Measurement errors in the theoretical model might exist, and further studies will need to explore more reliable measurements and results.

## Data Availability Statement

The original contributions presented in the study are included in the article/supplementary material, further inquiries can be directed to the corresponding author.

## Author Contributions

SS was contributed all to prepare the final version of the article and approved the submitted version.

## Conflict of Interest

The author declares that the research was conducted in the absence of any commercial or financial relationships that could be construed as a potential conflict of interest.
